# Avaliação Ativa de Sono e Depressão em Pacientes Idosos no Cenário da Cardiologia Ambulatorial: O Que Estamos Esperando?

**DOI:** 10.36660/abc.20210624

**Published:** 2021-09-01

**Affiliations:** 1 Faculdade de Medicina da Universidade de São Paulo Hospital das Clínicas da Faculdade Unidade de Hipertensão do Instituto do Coração do Hospital (InCor) São Paulo SP Brasil Unidade de Hipertensão do Instituto do Coração do Hospital (InCor) do Hospital das Clínicas da Faculdade de Medicina da Universidade de São Paulo, São Paulo, SP – Brasil

**Keywords:** Sono, Depressão;Idosos

O interesse nas relações entre distúrbios do sono, depressão e doença cardiovascular tem aumentado nas últimas décadas. A maioria das evidências disponíveis sugere que vários distúrbios do sono (incluindo privação de sono, insônia, e apneia obstrutiva do sono) não são somente meros espectadores, mas podem contribuir no aumento do risco cardiovascular.^1^^–^^3^ A prevalência e o impacto dos distúrbios do sono podem variar dependendo de algumas características clínicas (por exemplo: idade, sexo e comorbidades). Idade avançada é claramente um fator de risco para o desenvolvimento de distúrbios do sono; evidências consistentes mostraram um maior número de queixas associadas ao sono com o avançar da idade,^4^ incluindo insônia^5^ e apneia obstrutiva do sono.^2^^,^^3^ A redução no número de horas de sono e uma má qualidade do sono podem prejudicar vários domínios relacionados à qualidade de vida nos idosos.^4^^–^^6^ Ainda, a depressão está associada com várias consequências cognitivas e cardiovasculares.^7^ Com base na plausibilidade biológica que relaciona distúrbios do sono e depressão com risco cardiovascular,^7^^,^^8^ e a elevada prevalência de doenças cardiovasculares em idosos, é razoável afirmar que os distúrbios do sono contribuem para o alto risco cardiovascular observado nesse subgrupo de pacientes.

Nesta edição dos Arquivos de Brasileiro de Cardiologia, Lopes et al.^9^ apresentam um estudo prospectivo do tipo coorte com 160 pacientes idosos (entre 60 e 98 anos de idade) com o objetivo de estimar o risco de morte e de eventos cardiovasculares após oito anos de acompanhamento (de 2009 a 2017) naqueles que apresentaram insônia e sonolência excessiva durante o dia no basal. Sonolência excessiva durante o dia, qualidade do sono, e presença de sintomas depressivos foram avaliados por Escala de Sono de Epworth, Índice de Qualidade do Sono de Pittsburgh, e Escala de Depressão Geriátrica respectivamente.

Os desfechos cardiovasculares propostos avaliados oito anos depois (infarto do miocárdio, arritmias, valvulopatias, ataque isquêmico transitório e acidente vascular isquêmico ou hemorrágico) foram relatados pelos participantes ou seus familiares, e confirmados por revisão dos prontuários médicos nas respectivas unidades de saúde. Durante o seguimento, 40 mortes e 48 eventos cardiovasculares (incluindo eventos cerebrovasculares) ocorreram no período. Os autores observaram que os homens apresentaram um risco 88% maior de morte em comparação às mulheres. Além disso, depressão (RR=2,04; IC95%: 1,06-3,89), gravidade da insônia (RR=2,39; IC95% 1,52-4,56), e latência do sono de 16-30 minutos (RR=3,54; IC95% 1,26-9,94) e de 31-60 minutos (RR=2,23; IC 95%: 1,12-4,47) foram independentemente associados com risco aumentado de morte em idosos da comunidade. Os eventos cardiovasculares e cerebrovasculares foram preditos somente por hipertensão e diabetes. Os autores argumentaram que a maior mortalidade em homens está associada ao fato de eles apresentarem uma maior taxa de doenças crônicas, tais como doenças cardiovasculares. De fato, dados da população brasileira mostram a diferença na prevalência de doenças cardiovasculares e mortalidade entre os sexos, com maior taxa nos homens.^10^

Além disso, o presente estudo reforça o papel negativo de vários distúrbios do sono^2^^,^^3^ e depressão^11^^,^^12^ em pacientes idosos. Os mecanismos potenciais que ligam distúrbios do sono e depressão com mortalidade são múltiplos, incluindo mudanças na modulação do sistema nervoso autonômico, aumento da atividade simpática, inflamação sistêmica e aterogênese. Além disso, a ruptura em alguns sistemas de regulação cerebral, por exemplo, no eixo hipotálamo-pituitária-adrenal e na variação circadiana de cortisol e melatonina, pode contribuir para essa associação.^1^ A depressão está frequentemente associada com pobreza e baixo nível educacional, o que impõe limitações significativas no acesso aos serviços de saúde.^12^ Esses pacientes podem estar predispostos a uma adesão mais baixa a tratamentos crônicos, e a mudanças nos padrões de sono e no estilo de vida.^11^^,^^12^

As principais contribuições do estudo estão relacionadas ao delineamento prospectivo do estudo e a localidade da população estudada. O padrão de sono e o estado de depressão da população do nordeste brasileiro são pouco explorados. A elevada porcentagem de pobreza, baixo nível de educação, e vulnerabilidade social em comparação a outras regiões do país podem ter afetado os resultados do estudo, mas destacaram a necessidade de políticas de saúde direcionadas. Apesar dos pontos fortes do estudo, é importante discutirmos suas limitações. Primeiramente, esta é uma amostra relativamente pequena de pacientes para se avaliar desfechos duros, tais como morte e doença cardiovascular. Segundo, o delineamento observacional pode não capturar potenciais fatores residuais que possam contribuir para os desfechos propostos. Terceiro, as queixas relacionadas ao sono foram baseadas em relatos subjetivos. É razoável se esperar algum viés de memória e percepções errôneas sobre o sono em uma proporção significativa de pacientes. A disponibilidade de análises objetivas da duração do sono e da apneia obstrutiva do sono ajudaria a determinar o real impacto dos distúrbios do sono nos idosos.

Concluindo, o presente estudo reforça o potencial papel dos distúrbios do sono e da depressão na modulação da mortalidade em idosos. Esses resultados também destacam a necessidade de mais colaboração entre especialistas do sono e psiquiatras no manejo desses pacientes. Mais estudos abordando intervenções apropriadas a respeito desses fatores importantes contribuirão para avanços significativos nessa importante área de pesquisa.
