# Supplementary material for: Avaliação Ativa de Sono e Depressão em Pacientes Idosos no Cenário da Cardiologia Ambulatorial: O Que Estamos Esperando?
Source: Arq Bras Cardiol. 2021 Sep 1;117(3):455–6. [Article in Portuguese] doi: 10.36660/abc.20210624 (PMC8462956; doi:10.36660/abc.20210624)
Supplement: Translation

# Translation: Active Assessment of Sleep and Depression for elderly Patients in the Outpatient Cardiology Setting: What Are We Waiting for?

**Affiliations:** 1 São Paulo SP Brazil Unidade de Hipertensão do Instituto do Coração do Hospital (InCor) do Hospital das Clínicas da Faculdade de Medicina da Universidade de São Paulo, São Paulo, SP – Brazil

**Keywords:** Sleep, Depression, Elderly

The relationships between sleep disturbances, depression and cardiovascular disease have gained growing interest in the last decades. Most evidence available suggests that several sleep disturbances (including sleep deprivation, insomnia and obstructive sleep apnea) are not only innocent bystanders, but may contribute to increase the cardiovascular risk.^1^^–^^3^ The prevalence and impact of sleep disorders may vary depending on some clinical characteristics (for instance: age, gender and comorbidities). Aging is clearly a risk factor for developing sleep disturbances; consistent evidence have shown greater number of complaints associated with sleep with aging,^4^ including insomnia^5^ and obstructive sleep apnea.^2^^,^^3^ The reduction in the number of hours of sleep and poor sleep quality may impair multiple domains related to the quality of life in the elderly.^4^^–^^6^ Similarly, depression is associated with several cognitive and cardiovascular consequences.^7^ Based on the biological plausibility linking sleep disorders and depression with cardiovascular risk,^7^^,^^8^ and the high prevalence of cardiovascular diseases among elderly, it is reasonable to assume that sleep disorders add to the high cardiovascular burden observed in this subset of patients.

In this issue of the Arquivos de Brasileiro de Cardiologia, Lopes et al.^9^ reported a prospective cohort study of 160 elderly patients (between 60 and 98 years old) aiming to estimate the risk of death and cardiovascular events after eight years of follow-up (from 2009 to 2017) in those who presented insomnia and excessive daytime sleepiness detected at baseline. Excessive daytime sleepiness, sleep quality and presence of depressive symptoms were evaluated by the Epworth Sleepiness scale, the Pittsburgh Sleep Quality Index and the Sheikh and Yesavage Geriatric Depression Scale, respectively.

The proposed cardiovascular endpoints evaluated eight years later (myocardial infarction, arrhythmias, valvulopathies, transient ischemic attack and ischemic or hemorrhagic stroke) were self-reported by the participants or their families, and confirmed by reviewing the medical records in the respective health centers. Deaths were adjudicated using death certificates. In the follow-up, 40 deaths and 48 cardiovascular events (including cerebrovascular events) occurred in the period. The authors found that men had an 88% increased risk of death as compared to women. In addition, depression (RR=2.04; 95% CI: 1.06-3.89), the severity of insomnia (RR=2.39; 95% CI: 1.52-4.56), and sleep latency of 16-30 minutes (RR=3.54; 95% CI: 1.26-9.94) and 31-60 minutes (RR=2.23; 95% CI: 1.12-4.47) were independently associated with increased risk of death in community-dwelling elderly. Non-fatal cardiovascular and cerebrovascular events were predicted by hypertension and diabetes only. The authors argued that the association of higher mortality in men is associated with the fact that they have a higher rate of chronic diseases, such as cardiovascular diseases. Consistently, data from the Brazilian population demonstrate the difference in the prevalence of cardiovascular disease and mortality between genders, with a higher rate in men.^10^

Additionally, the current study reinforces the negative role of several sleep disturbances^2^^,^^3^ and depression^11^^,^^12^ in elderly patients. The potential mechanisms connecting sleep disturbances and depression with mortality are multiple including changes in autonomic nervous system modulation, increased sympathetic activity, systemic inflammation and atherogenesis. Moreover, disruption of some brain regulatory systems, for example hypothalamic– pituitary–adrenal axis and circadian variation of cortisol and melatonin, may contribute to this association.^1^ Depression is frequently associated with poverty and low education level, which impose significant limitations in the access to health services.^12^ These patients may be predisposed to lower adherence to chronic treatments, and changes in sleep patterns and lifestyle.^11^^,^^12^

The major contributions of the current investigation are related to the prospective study design and the location of the study population. The sleep patterns and depression status of the Northeast Brazilian population are poorly explored. The high percentage of poverty, low education and social vulnerability in comparison to other regions in Brazil may have affected the results, but underscored the need of dedicated health policies. Despite the strengths, it is important to discuss limitations of the current study. First, this is a relative small sample of patients to evaluate hard endpoints such as death and cardiovascular disease. Second, the observational design may not capture potential residual factors that might help to contribute to the proposed endpoints; third, the sleep complaints were based on subjective reports. It is reasonable to expect some recall bias and sleep misperceptions in a significant proportion of patients. Ideally, the availability of objective analysis of sleep duration and obstructive sleep apnea would be helpful in determining the real impact of sleep disturbances in the elderlies.

In conclusion, the current study reinforces the potential role of sleep disturbances and depression in modulating mortality in the elderly. These results also underscore the need for more collaboration among sleep specialists and psychiatrists for managing these patients. Future investigations addressing appropriate interventions on these important factors will be helpful for significant advances in this important research area.
